# The association between cognitive function and white matter lesion location in older adults: a systematic review

**DOI:** 10.1186/1471-2377-12-126

**Published:** 2012-10-30

**Authors:** Niousha Bolandzadeh, Jennifer C Davis, Roger Tam, Todd C Handy, Teresa Liu-Ambrose

**Affiliations:** 1 Department of Physical Therapy, University of British Columbia, 212-2177 Wesbrook Mall, Vancouver, BC V6T 1Z3, Canada; 2 Centre for Clinical Epidemiology and Evaluation, University of British Columbia, 212-2177 Wesbrook Mall, Vancouver, BC V6T 1Z3, Canada; 3 Department of Radiology, University of British Columbia, 212-2177 Wesbrook Mall, Vancouver, BC V6T 1Z3, Canada; 4 Department of Psychology, University of British Columbia, 212-2177 Wesbrook Mall, Vancouver, BC V6T 1Z3, Canada; 5 Brain Research Centre, University of British Columbia, 212-2177 Wesbrook Mall, Vancouver, BC V6T 1Z3, Canada; 6 Centre for Hip Health and Mobility, Vancouver Coastal Health Research Institute, 212-2177 Wesbrook Mall, Vancouver, BC V6T 1Z3, Canada

**Keywords:** White matter lesions, Distribution, Cognition, Aging

## Abstract

**Background:**

Maintaining cognitive function is essential for healthy aging and to function autonomously within society. White matter lesions (WMLs) are associated with reduced cognitive function in older adults. However, whether their anatomical location moderates these associations is not well-established. This review systematically evaluates peer-reviewed evidence on the role of anatomical location in the association between WMLs and cognitive function.

**Methods:**

In accordance with the preferred reporting items for systematic reviews and meta-analysis (PRISMA) statement, databases of EMBASE, PUBMED, MEDLINE, and CINAHL, and reference lists of selected papers were searched. We limited our search results to adults aged 60 years and older, and studies published in the English language from 2000 to 2011. Studies that investigated the association between cognitive function and WML location were included. Two independent reviewers extracted: 1) study characteristics including sample size, sample characteristic, and study design; 2) WML outcomes including WML location, WML quantification method (scoring or volume measurement), strength of the MRI magnet in Tesla, and MRI sequence used for WML detection; and 3) cognitive function outcomes including cognitive tests for two cognitive domains of memory and executive function/processing speed.

**Results:**

Of the 14 studies included, seven compared the association of subcortical versus periventricular WMLs with cognitive function. Seven other studies investigated the association between WMLs in specific brain regions (e.g., frontal, parietal lobes) and cognitive function. Overall, the results show that a greater number of studies have found an association between periventricular WMLs and executive function/processing speed, than subcortical WMLs. However, whether WMLs in different brain regions have a differential effect on cognitive function remains unclear.

**Conclusions:**

Evidence suggests that periventricular WMLs may have a significant negative impact on cognitive abilities of older adults. This finding may be influenced by study heterogeneity in: 1) MRI sequences, WML quantification methods, and neuropsychological batteries; 2) modifying effect of cardiovascular risk factors; and 3) quality of studies and lack of sample size calculation.

## Background

The world’s population is aging [1]. Maintaining cognitive function is essential for healthy aging and to function autonomously within society.

With age, the brain undergoes both structural and functional changes [2-5]. Specifically, cerebral white matter lesions (WMLs) are prevalent among adults aged 60 years or older [6,7]. These lesions are due to damage to the brain parenchyma [8], ranging from demyelination to complete axonal disruptions [9,10]. Although their pathogenesis is unknown, there is a growning recognition that WMLs are most likely the result of cerebrovascular disorders and cerebral ischemia [8,11-13]. The current gold standard for diagnosis of WMLs includes various MRI sequences, such as T1, T2, proton density (PD), or fluid attenuated inversion recovery (FLAIR).

White matter lesions are associated with both impaired mobility and reduced cognitive performance as measured by standard neuropsychological testing, which might be caused by impairing the speed or integrity of signal transmission [14,15]. Specially, WML load has a negative impact on multiple domains of cognitive function such as memory, processing speed, attention, and executive function [8,16]. Pantoni et al. [16] summarized the results of 16 studies focusing on the effect of WMLs on different cognitive domains. Their results showed that, despite the fact that the probability of finding a positive association between WML load and cognitive decline may be affected by the cognitive domains assessed, an effect of WML on cognition was present invariably. However, emerging evidence suggests that WML distribution, as well as load, may also be a predictor of reduced cognitive performance [17,18]. In a study by Kim et al. [17], it is suggested that a specific distribution of fiber tract damage is more associated with cognitive and motor impairment, compared with the total WML load. Thus, we conducted a systematic review to ascertain the role of anatomical location in the association between WMLs and cognitive function in older adults.

## Methods

### Search strategy

**Figure 1:**
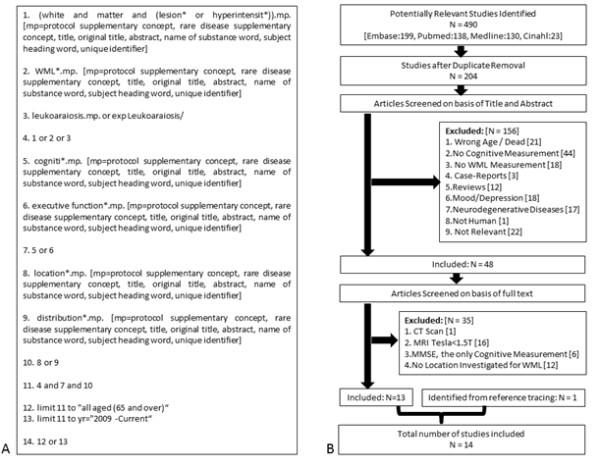
(A): Searching strategy retrieved from Ovid, (B): Flowchart of study selection.

In accordance with the preferred reporting items for systematic reviews and meta-analysis (PRISMA) statement [19], we [NB, JCD and TLA] conducted a search of EMBASE, MEDLINE, PUBMED, and CINAHL supplemented by manual search of included articles’ reference lists. The search strategy (Figure 1(A)) was developed by April 19^th^ 2011, and includes studies from 2000 to 2011. We limited our search results to adults aged 60 years and older, and studies published in the English language.

### Study selection

We excluded case-studies, reviews, and articles lacking WML quantification or measurements of cognitive function, based on their titles and abstracts (Figure 1(B)). Also, any study with the primary focus on psychiatric conditions (e.g., depression) or progressive neurodegenerative diseases (except for Alzheimer’s disease (AD) and cerebrovascular disorders due to the high prevalence of WMLs) was excluded. Based on full text review, we excluded studies that: 1) used computed tomography (as it is less sensitive than MRI in detection of WMLs [20]), or used MRI device with a magnet strength of less than 1.5T and; 2) assessed only global cognition (measured by mini-mental state examination (MMSE)) as it may not be sensitive to the differential effects of WML location; and 3) did not detail WML location.

### Data extraction and quality assessment

We [NB and TLA] developed a list of extraction items including: 1) study characteristics; 2) WML outcomes; and 3) cognitive function outcomes. One study [21] did not report the strength of MRI magnet and NB contacted the author.

Two authors [NB, TLA] independently evaluated each study based on four quality assessments questions (see Table 1), and all the discrepancies were reviewed by JCD and RT. Assessing the validity of WML quantification was influenced by the difficulty in the differential diagnosis of WMLs, which requires expert radiological knowledge to be done accurately [22]. In addition, the intensity range of lesions typically overlaps with those of healthy tissues, so automatic identification methods tend to produce more false positives as compared with manual identification by a radiologist [23]. Therefore, our assessment favors quantification methods that use radiologist/physician identification of WMLs. We used dichotomized answers (+: yes, -: no) for the quality assessment questions.

**Table 1:** Quality assessment results for included studies

**Reference**	**Q1. Was the WML identification done by a radiologist/physician?**	**Q2. Was the cognitive performance measured using a standardized method?**	**Q3. Was there a sample size calculation?**	**Q4. Were age or education considered as confounders?**
Groot et al. et al. [24]	+	+	-	+
Shenkin et al. [25]	+	+	-	-
Baune et al. [26]	-	+	-	+
Kim et al. [27]	-	+	-	+
Silbert et al. [28]	-	+	-	+
McClleland et al. [21]	+	+	-	+
Wright et al. [29]	-	+	-	+
Kaplan et al. [30]	-	+	-	+
Wakefield et al. [31]	-	+	-	+
O’Brien et al. [32]	+	+	-	+
Smith et al. [14]	-	+	-	+
Burns et al. [33]	+	+	-	+
Ishii et al. [34]	+	+	-	+
Tullberg et al. [35]	-	+	-	-

## Results

### Overview of studies

The initial number of articles identified was 490 (Figure 1(B)). After duplicate removal, 156 papers were further excluded using their title and abstract. We conducted a full text review of the remaining 48 articles. In total, 14 articles met the inclusion criteria (see Tables 2, 3, 4, 5). These articles were further categorized into two groups based on the cognitive status of their study samples: 1) studies that did not compare subjects based on cognitive status (i.e., normal, cognitively impaired but not demented, and demented); and 2) studies that classified and compared subjects based on cognitive status. Table 6 shows the most commonly-used cognitive tests in the 14 included studies.

**Table 2:** Characteristics of studies included in this systematic review

**Reference**	**Sample size**	**Study design**
**Publishing year**	**Sample characteristics**	
Groot et al. [24]	1077	Cross-Sectional
2000	Subsample of Rotterdam and Zeotemeer Studies	
Shenkin et al. [25]	105	Cross-Sectional
2005	Random Sample of Community-Dwelling Participants	
Baune et al. [26]	268	Cross-Sectional
2009	Subsample of MEMO Study	
Kim et al. [27]	84	Cross-Sectional
2011	Random Sample of Normals/Recruited from Memory Clinic	
Silbert et al. [28]	104	Longitudinal
2008	Subsample of Oregon Brain Aging Study	
McClleland et al. [21]	3647	Cross-Sectional
2000	Subsample of CHS Cohort	
Wright et al. [29]	656	Cross-Sectional
2008	Subsample of NOMAS Cohort study	
Kaplan et al. [30]	95	Cross-Sectional
2009	Random Sample of Participants	
Wakefield et al. [31]	99	Cross-Sectional
2010	Sample Selected for a Longitudinal Study	
O’Brien et al. [32]	149	Cross-Sectional
2002	Subsample of SCOPE Study	
Smith et al. [14]	145	Cross-Sectional
2011	Subsample of Prospective Study	
Burns et al. [33]	156	Cross-Sectional
2005	88 Normal (CDR=0), 68 Early-Stage AD (CDR=0.5,1)	
Ishii et al. [34]	453	Cross-Sectional
2007	340 (CDR=0), 113 (CDR=0.5)	
Tullberg et al. [35]	78	Cross-Sectional
2004	22 Normal (CDR=0), 30 CIND (CDR=0.5), 26 Demented (CDR≥1)	

Abbreviations: *MEMO* = Memory and Morbidity in Augsburg Elderly; *CDR* = Clinical Dementia Rating Scale; *CHS* = Cardiovascular Health Study; *NOMAS* = Northern Manhattan Study; *SCOPE* = Study on Cognition and Prognosis in Elderly; *CIND* = Cognitively Impaired not Demented.

**Table 3:** Outcome Measures: white matter lesion quantification

**Reference**	**Sequence**	**WML Location**	**MRI Magnet**
**WML Type**	**WML Quantification**		
Groot et al. [24]	PD, T1, T2	S: Four lobes of Frontal, Parietal, Occipital, and Temporal	1.5 T
P, S, Regions	Scoring	P: Adjacent frontal horns, lateral ventricles wall, and occipital horns	
Shenkin et al. [25]	T2, FLAIR	-	1.5 T
S, P	Scoring		
Baune et al. [26]	PD, T1, T2	-	1.5 T
S, P	Scoring		
Kim et al. [27]	T2, FLAIR	-	1.5 T
S, P	Volum		
Silbert et al. [28]	PD, T2	-	1.5 T
S, P	Volume		
McClleland et al. [21]	PD, T1, T2	Cerebral White Matter, Cerebellar White Matter, Basal Ganglia	1.5 T
Regions	Scoring		
Wright et al. [29]	PD, T2, FLAIR	Frontal, Deep, and Occipital-Temporal-Parietal	1.5 T
S, I, Regions	Volume		
Kaplan et al. [30]	T2, FLAIR	Frontal and Posterior Regions	3.0 T
Regions	Volume		
Wakefield et al. [31]	T1, FLAIR	Anterior, Superior, Posterior Corona Radiata	3.0 T
Regions	Volume	Cingulate Gyrus, Genu, Body, Splenium of Corpus Callusum	
		Anterior and Posterior Limb of Internal Capsule	
		Superior Longitudinal Fasciculus	
O’Brien et al. [32]	T2, FLAIR	Internal and External Capsule	1.5 T
Regions	Scoring		
Smith et al. [14]	PD, T1, T2	Whole Brain	1.5 T
Regions	Volume		
Burns et al. [33]	T1, T2	S: Frontal, Parietal, Temporal, and Occipital Lobes	1.5 T
S, P, Regions	Scoring	P: Right and Left Frontal Horns, Posterior Horns, and Ventricular Bodies	
Ishii et al. [34]	T2	S: Left and Right	1.5 T
P, S, Regions	Scoring	P: Anterior and Posterior	
Tullberg et al. [35]	T1, T2	Orbitofrontal, Prefrontal, Dorsolateral Frontal, Parietal, and Occipitotemporal	1.5 T
Regions	Volume		

Abbreviations: *PD* = Proton Density; *FLAIR* = Fluid Attenuated Inversion Recovery.

**Table 4:** Outcome measures: Cognitive tests used for two cognitive domains of memory and executive function/processing speed

**Reference**	**Executive function / Processing speed**	**Memory**
Groot et al. et al. [24]	Stroop, Letter-Digit Substitution Task, Verbal Fluency	Rey’s Auditory, Memory Scanning Task
Shenkin et al. [25]	Verbal Fluency, Controlled Word Association, Moray House Test, Raven’s Progressive Matrices	Wechsler Memory Scale
Baune et al. [26]	Stroop, Letter-Digit Substitution Task	3-Word Recall
Kim et al. [27]	Boston Naming, Buccofacial Praxis Test, Semantic Controlled Oral Word Association Test, Stroop Color, Word Test	Seoul Verbal Learning Test, Ray Complex Figure Test, Delayed Recall and Recognition, Digit Span Tests
Silbert et al. [28]	-	Delayed Story Recall
McClleland et al. [21]	Digit-Symbol Substitution Task	-
Wright et al. [29]	Color Trail 1 & 2	-
Kaplan et al. [30]	Stroop, Trail Making, CalCap	Repeated Battery for Neuropsychological Status
Wakefield et al. [31]	Stroop, Trail Making 1 & 2, CalCap	-
O’Brien et al. [32]	Verbal Fluency, Trail Making 1 & 2	Memory Component of CDR
Smith et al. [14]	Letter Fluency, Trail Making 2	Episodic Memory, Alpha Span Test
Burns et al. [33]	Trail Making 1 & 2, Short Blessing Test, Boston Naming	Wechsler Memory Scale, Wechsler Adult Intelligence Scale
Ishii et al. [34]	Verbal Fluency, Trail Making Test, Benton’s Visual Form Test	ADAS-Cog, 10 Word Recall, Digit Span Forward
Tullberg et al. [35]	Verbal Fluency	Wechsler Memory Scale, Word List Learning, Digit Span Backward

Abbreviations: *CalCap* = California Computerized Assessment Package; *CDR* = Clinical Dementia Rating Scale; *ADAS-Cog* = Alzheimer’s Disease Assessment Scale-Cognitive.

**Table 5:** Association between the structural location of white matter lesion (i.e., subcortical, periventricular, or regional) with two domains of cognitive function (i.e., memory and executive function/processing speed)

**Reference**	**Association**
Groot et al. et al. [24]	Controlled for subcortical, periventricular WMLs were associated with memory and executive function/processing speed.
Shenkin et al. [25]	Subcortical and periventricular WMLs were not associated with any of the cognitive measurements.
Baune et al. [26]	Subcortical WMLs were associated with memory.
	As a subgroup of subcortical WMLs, infarction lesions were associated with executive function/processing speed.
	Periventricular WMLs were not associated with any of the cognitive functions.
Kim et al. [27]	Only periventricular WML was significantly correlated with memory and executive function/processing speed, when both the periventricular and subcortical WMLs were entered simultaneously into the regression model.
Silbert et al. [28]	Change in subcortical WMLs (excluding infarction lesions) was associated with memory decline. This association was not true for periventricular WMLs.
McClleland et al. [21]	White matter lesions were associated with executive function/processing speed, in all white matter regions of cerebrum, cerebellum, and basal ganglia.
Wright et al. [29]	Subcortical WMLs (including infarction lesions) were associated with executive function/processing speed, in regions of frontal and deep white matter.
Kaplan et al. [30]	White matter lesions were associated with memory and executive function/processing speed, in frontal regions.
Wakefield et al. [31]	White matter lesions were associated with executive function/processing speed in white matter regions of posterior corona radiata and splenium of corpus callosum.
O’Brien et al. [32]	White matter lesions were associated with speed of memory retrieval and executive function/processing speed.
Smith et al. [14]	White matter lesions were associated with memory and executive function/processing speed. White matter lesions in the following locations were significantly associated with memory: right inferior temporal-occipital, left temporal-occipital periventricular, and right parietal periventricular white matter; and anterior limb of internal capsule. Also, WMLs in the following regions were significantly associated with executive function: the bilateral inferior frontal, temporal-occipital periventricular, right parietal periventricular, and prefrontal white matter; and the anterior limb of the internal capsule bilaterally.
Burns et al. [33]	For non-demented participants, only associate memory was associated with periventricular WMLs. For participants with early-stage Alzheimer’s Disease (AD), memory and executive function/processing speed were associated with both periventricular and subcortical WMLs.
Ishii et al. [34]	For CDR=0 group, anterior periventricular WML and a test of executive function/processing speed were significantly correlated.
Tullberg et al. [35]	In non-demented individuals, increased volumes of frontal (specifically prefrontal and dorsolateral), parietal, and occipital WML were separately associated with lower executive function/processing speed scores.
	Frontal WMLs were also associated with reduced memory function in non-demented group. No association was found for individuals with dementia.

Abbreviations: *WML* = White Matter Lesion; *CDR* = Clinical Dementia Rating Scale.

**Table 6:** The most commonly-used neuropsychological tests in the included studies

	
Executive Function	Trail-Making Test, Stroop Test, Verbal Fluency Test
Memory	Wechseler Memory Scale, Word Recall Test

### Studies that did not compare subjects based on cognitive status

#### Subcortical vs. periventricular WML

Five studies [24-28] – four cross-sectional studies and one prospective study -- compared the association of subcortical versus periventricular WMLs with cognitive function. In the first cross-sectional study of 1077 older adults [24], WMLs were defined as T2 and PD hyperintensities that were not T1 hypointensities. Four lobes of frontal, parietal, occipital, and temporal were considered for subcortical WML scoring. Three regions adjacent to frontal horns, lateral ventricles wall, and occipital horns were selected for periventricular WML scoring. The neuropsychological battery evaluated two domains of memory and executive function/processing speed. The results showed that when controlled for subcortical WML severity, increased periventricular WML severity in all the three regions was associated with reduced performance in both cognitive domains (p<0.01). However, when controlled for periventricular WMLs, no such association was found for subcortical WMLs.

In the second cross-sectional study of 105 older adults [25], WMLs were identified using T2 and FLAIR scans. Results showed that higher periventricular and subcortical WML scores were not significantly associated with reduced memory and executive function/processing speed.

In a sample of 268 older adults [26], WMLs were categorized into three groups of: 1) large subcortical WMLs defined as PD and T2 hyperintensities that were not T1 hypointensities; 2) infarction lesions defined as lesions of ≥2 mm that were either T2 hyperintensities, or PD and T1 hypointensities; and 3) periventricular WMLs. The results indicated that large subcortical WMLs were significantly associated with memory, and infarction lesions were significantly associated with executive function/processing speed (p<0.05). Contrary to the results of two previously mentioned studies, this study found no significant relationship between periventricular WMLs and cognitive performance.

In the last cross-sectional study, Kim et al. [27] defined WMLs as T2 and FLAIR hyperintensities. Over the 84 older adults, only periventricular WML was significantly correlated with memory and executive function/processing speed, when both the periventricular and subcortical WMLs were entered simultaneously into the regression model (p<0.05).

The one longitudinal study [28] used a sample of 104 subjects to investigate the impact of WML volume progression on the rate of cognitive decline. White matter lesions were defined as PD and T2 hyperintensities. Infarction lesions – detected by their clean or sharp edges, and if they were relatively dark on PD scans – were excluded from WML analysis. The neuropsychological battery assessed only memory. Higher rate of subcortical (but not periventricular) WML volume change was associated with increased rate of decline in memory scores (p<0.001).

#### Regional WMLs

Six cross-sectional studies [14,21,29-32] examined the association between WMLs in specific brain regions (e.g., frontal, parietal, etc.) and cognitive performance. McClelland et al. [21] defined WMLs as PD and T2 hyperintensities that were T1 hypointensity. The results in 3647 older adults suggested that WMLs located in cerebellar and cerebral white matter and basal ganglia were significantly associated with reduced processing speed performance (p<0.05).

Among 656 older adults, Wright et al. [29] differentiated subclinical infarction lesions from the rest of WMLs based on the size, location, and imaging characteristics obtained from PD, T2, and FLAIR scans. They were grouped by location into frontal, deep and occipital-temporal-parietal networks. The neuropsychological battery assessed only executive function/processing speed. The results demonstrated that individuals with infarction lesions in frontal and deep locations had significantly worse cognitive performance (p<0.05).

Kaplan et al. [30] studied a sample of 95 older adults. White matter lesions were defined as FLAIR and T2 hyperintensities, and were categorized into frontal and posterior regions. The results showed that frontal WMLs were associated with memory (p<0.05) and executive function/processing speed (p<0.001).

Furthermore, Wakefield et al. [31] detected WMLs based on FLAIR and T1 scans in a sample of 99 community-dwelling older adults. The following regions of interest were segmented for WMLs: anterior, superior, and posterior corona radiata; cingulate gyrus, genu, body, and splenium of corpus callusum; anterior and posterior limb of internal capsule; and superior longitudinal fasciculus. The neuropsychological battery assessed only executive function/processing speed. In regions of posterior corona radiata and splenium of corpus callosum, the total amount of WMLs was significantly associated with executive function/processing speed (p<0.05).

O’Brien et al. [32] detected WMLs based on FLAIR and T2 scans, in 149 older adults. The focus of their analysis was on the distribution of WMLs in the internal and external capsule. They found that WMLs from both regions were significantly associated with cognitive performance of speed of memory retrieval and executive function/processing speed (p<0.05).

Smith et al. [14] analyzed WML distribution using PD, T2, and T1 scans in the whole brain of 147 older adults. The total volume of WMLs was associated with the cognitive performance of memory (p<0.01) and executive function (p=0.05). In the following locations, WMLs were significantly associated with memory: right inferior temporal-occipital, left temporal-occipital periventricular, and right parietal periventricular; and anterior limb of internal capsule. Also, WMLs in the following regions were significantly associated with executive function: the bilateral inferior frontal, temporal-occipital periventricular, right parietal periventricular, and prefrontal white matter; and the anterior limb of the internal capsule bilaterally.

### Studies that classified and compared subjects based on cognitive status

#### Subcortical vs. periventricular WML

Among the studies that classified participants based on their cognitive status, two cross-sectional studies [33,34] compared the effects of subcortical and periventricular WMLs. Burns et al. [33] included 88 non-demented participants (clinical dementia rating (CDR) score=0), 68 with early-stage AD (48 with very mild AD (CDR=0.5), and 20 with mild AD (CDR=1)). White matter lesions were defined as T2 hyperintensities that were T1 hypointensities. Subcortical WMLs were rated in regions of frontal, parietal, temporal, and occipital lobes. Periventricular WMLs were rated in right and left frontal horns, posterior horns, and ventricular bodies. For non-demented participants, only associate memory was associated with periventricular WMLs (p<0.01). For participants with early-stage AD, memory and executive function/processing speed were associated with both periventricular and subcortical WMLs (p<0.05).

Ishii et al. [34] detected WMLs based on T2 hyperintensities. Sample of 453 older adults were categorized into two groups of CDR=0 and CDR=0.5. Anterior and posterior periventricular WMLs, as well as left and right subcortical WMLs were segmented. The results suggested that, for CDR=0 group, anterior periventricular WMLs and a test of executive function/processing speed were significantly correlated (p=0.001).

#### Regional WML

The last study [35] detected WMLs based on T1 and T2 scans. They categorized 78 older adults into three cognitive groups: normal (CDR=0), cognitively impaired but not demented (CDR=0.5), and demented (CDR≥1), either by AD or vascular dementia. WMLs were analyzed in regions of orbitofrontal, prefrontal, dorsolateral frontal, parietal, and occipitotemporal. In non-demented individuals, increased volumes of frontal (specifically, prefrontal and dorsolateral), parietal, and occipital WML were separately associated with lower executive function/processing speed scores (p<0.05). Frontal WMLs were also associated with reduced memory function in non-demented group (p<0.05). No association was found for individuals with dementia.

### Quality assessment

The quality assessment results for each of the four questions are presented in Table 1: 1) in seven studies, WML identification is done by a radiologist/physician, while the remaining used automatic methods; 2) all the articles employed standard methods for cognitive assessment; 3) none of the studies provided sample size calculation; and 4) the statistical analyses of twelve studies included age or education as confounders.

## Discussion

### Subcortical vs. periventricular WMLs

Based on their proximity to ventricles, WMLs were classified as subcortical or periventricular in seven studies [24-28,33,34]. The results show that more studies have found an association between periventricular WMLs with the cognitive domain of executive function, than subcortical WMLs.

Subcortical WMLs are believed to primarily disrupt short connections, and thus impairing cognitive performance supported by the specific brain region [24]. For example, dexterous hand and arm movements are generally thought to be primarily supported by the motor cortex. Therefore, subcortical WMLs in this specific region can result in reduced performance in hand and arm dextrous movements [36]. In contrast, periventricular WMLs disrupt longer connections to spatially distant cortical areas, and thus can cause cognitive performance decline in multiple domains [24,27]. For example, executive function tasks typically used in research experiments depend on multiple brain regions (i.e., frontal and non-frontal) which are not necessarily located spatially close to each other [37]. Therefore, any disruption in long white matter tracts traversing from periventricular areas may initially reduce the axonal transmission speed [38], and later cause impaired executive function. In summary, cognitive function depends on intact connections within subcortical areas and between cortical and subcortical structures, and any disruption in these connections may impair cognitive function.

We categorized all included studies into two major cognitive domains which are sensitive to aging: 1) memory; or 2) executive function/processing speed. The latter category was a combination of two cognitive domains based on the idea that they are not mutually exclusive, and one needs to control for their mutual relationship before examining their unique effects [39].

For memory, out of seven studies, three studies [24,27,33] found a significant association between *periventricular* WMLs and memory performance, two studies [26,28] found a significant association between *subcortical* WMLs and memory performance, and two studies [25,34] did not find any association.

For executive function/processing speed, out of six studies, three studies [24,27,34] found a significant association between *periventricular* WMLs and executive function/processing speed, while only one study [26] found a significant association between *subcortical* WMLs and executive function/processing speed. Two studies [25,33] did not find any association.

Thus, our overall results show that greater number of studies found an association between cognitive impairment (in both domains of memory and executive function/processing speed) and periventricular WMLs, compared with subcortical WMLs. Moreover, greater number of studies showed an association between impairment in the domain of executive function/processing speed with periventricular WMLs, compared to subcortical WMLs.

As highlighted earlier, periventricular WMLs may impact multiple domains of cognition because they disrupt distant connections. Hence, our findings concur with the general knowledge that the domain of executive function/processing speed may depend on multiple brain regions and spatially distant connections [37,40].

### Regional WML

Seven studies [14,21,29-32,35] investigated regional WMLs. No common pattern was evident secondary to the heterogeneity of regions studied.

The following regions demonstrated significant associations between WMLs and cognitive function: cerebral white matter, cerebellar white matter, and basal ganglia [21]; frontal (dorsolateral frontal and prefrontal) [29-31], parietal, occipital, and temporal lobes [29,31,35]; internal and external capsule [32]; posterior corona radiata, and splenium corpus callosum [31]. This systematic review provides researchers with a summary set of brain regions in which an association have been found between WMLs and cognitive performance. To better understand the role of anatomical location in the association between WML and cognitive function, future studies should examine the spatial distribution of WMLs on the whole brain, or specific set of brain regions identified in this review as being highly associated with cognitive dysfunction.

### Limitations

The discrepancies between the results may be due to the heterogeneous study methodologies and the quality of included studies.

#### Different MRI sequences, WML quantification methods, and neuropsychological batteries

The included studies were heterogeneous in MRI sequences for WML detection (i.e., PD, T1, T2, or FLAIR), WML quantification method (i.e., scoring or volume measurements), and components of neuropsychological batteries. This likely contributed to variability in our results.

Moreover, two different methods were used for WML quantification: 1) scoring [24,25,33,34]; and 2) volume measurement [26-28]. Scoring measures are usually done manually, and show a higher accuracy for selection of subtle WMLs, compared to automatic volumetric methods. However, these methods vary significantly in terms of lesion classification and severity scoring. Moreover, each scoring method has its own specific limitations.

For WML volume measurement, there are two steps. The first step is identifying lesions, which can be done either manually by an expert radiologist or automatically. After the WMLs are identified manually or automatically, one can proceed to the second step, which is measuring WML volumes automatically. It has been shown that both scoring and volumetric quantification methods are reliable for measuring WML load [41,42]. However, periventricular and subcortical WMLs quantified by these two quantification methods are differently associated with cognitive function [42]. Out of three studies which used *volume measurement*, two studies [26,28] showed a significant association between the *subcortical* WMLs and cognitive performance. Out of four studies which used *scoring*, three [24,33,34] showed a significant association between *periventricular* WMLs and cognitive performance. These results suggest that *scoring* might have biased the results toward *periventricular* WMLs. Conversely, *volume measurement* might be problematic for *periventricular* WMLs due to their similar appearance to CSF on some MRI sequences (e.g., T2 or T1) [43].

#### Modifying effect of cardiovascular risk factors

There is a growing recognition that WMLs are associated with age and cardiovascular risk factors [8,44]. However, all but one included study [21] considered the modifying effect of cardiovascular risk factors in the statistical analysis. We recommend that future studies consider including cardiovascular risk factors in their analysis.

#### Quality of studies and lack of sample size calculation

One study [25] did not demonstrate a significant association between any type of WMLs and any of the cognitive tasks. Based on our quality assessment, this study is the only study categorizing WMLs locations as subcortical and periventricular that did not consider age or education as potential confounders. Therefore, we concluded that this study did not provide strong evidence for the lack of correlation between WMLs and cognitive function.

Moreover, the lack of sample size calculations in all of the included studies might have resulted in possible type II errors. However, we do recognize that the lack of sample size calculations may be due to the dearth of data in this research area [45].

## Conclusions

This study provides the first in depth analysis of brain regions where an association between WML location and cognitive decline has been found in older adults. Specifically, studies that considered periventricular versus subcortical WMLs suggest that, compared with subcortical WMLs, periventricular WMLs may have a greater negative impact on cognitive performance. Moreover, periventricular WMLs appear to be more associated to the domain of executive function/processing speed, than to the domain of memory. To further clarify the association of cognitive function with WML locations, we suggest that future studies consider spatial distribution of WMLs on the whole brain.

We did not proceed to a meta-analysis of the results, primarily because of the small number of studies systematically found on this topic. Moreover, the neuropsychological batteries used for assessing cognitive status, the WML quantification method, and MRI sequences used for WML detection varied vastly between studies. Thus, it was not feasible to conduct a meta-analysis.

### Source of funding

This work was supported by the Canadian Stroke Network, the Heart and Stroke Foundation of Canada, and the Canadian Institutes of Health Research (MOB-93373) to TLA. TLA is a Canada Research Chair (Tier II) in Physical Activity, Mobility, and Cognitive Neuroscience, a Michael Smith Foundation for Health Research Scholar, a Canadian Institutes of Health Research New Investigator, and a Heart and Stroke Foundation of Canada's Henry JM Barnett's Scholarship recipient. JCD is a Michael Smith Foundation for Health Research Post-Doctoral Fellow and a Canadian Institutes of Health Research Post-Doctoral Fellow. NB is a Heart and Stroke Foundation of Canada Doctoral Trainee.

## Competing interests

The authors declare that they have no competing interests.

## Authors’ contribution

All authors participated, read, and approved the final manuscript.

## Pre-publication history

The pre-publication history for this paper can be accessed here:

http://www.biomedcentral.com/1471-2377/12/126/prepub
